# Opening the Black Box of Ayres Sensory Integration^®^: Clarifying Mechanisms of Change from Treatment Theory to Participation

**DOI:** 10.3390/brainsci16101131

**Published:** 2026-10-20

**Authors:** Stacey Reynolds, Lauren Andelin, Caterina LaRocca

**Affiliations:** Department of Occupational Therapy, Virginia Commonwealth University, Richmond, VA 23227, USA; akselrodls@vcu.edu (L.A.); laroccac@vcu.edu (C.L.)

**Keywords:** sensory integration, rehabilitation treatment specification system, reporting guideline for intervention description in rehabilitation, data-driven decision making, treatment theory, enablement

## Abstract

**Highlights:**

**What are the main findings?**

- It is essential to clarify the distinction between treatment and enablement theories within clinical practice and education to strengthen clinical reasoning.
- Sensory Integration theory, as described by Ayres, is best understood as a treatment theory.

**What are the implications of the main findings?**

- Improved integration of treatment theory, enablement theory, implementation frameworks, and reporting guidelines may strengthen the translation of neuroscience into rehabilitation practice.
- Applying Sensory Integration theory as a treatment theory will enhance learners’ ability to understand what intervention strategies should be selected, and why these strategies are expected to influence brain function and behavior.

**Abstract:**

Ayres Sensory Integration^®^ (ASI) remains one of the most widely used and debated intervention approaches in occupational therapy and related disciplines. Recent advances in rehabilitation treatment specification and reporting frameworks, including the Rehabilitation Treatment Specification System (RTSS) and the Reporting Guideline for Intervention Description in Rehabilitation (GUIDE-Rehab), emphasize the need for precise articulation of treatment ingredients, mechanisms of action, and targets. However, interventions grounded in neurodevelopmental theories, such as Ayres’ theory of sensory integration, are complex, and may present challenges when interpreted within linear causal models. This paper argues that ASI theory is best understood as a treatment theory targeting proximal sensory–motor capacities, while participation-level outcomes may be more comprehensively explained through an enablement framework. Integrating this perspective with data-driven decision making (DDDM) clarifies longstanding ambiguities in the ASI literature and strengthens intervention specification, evaluation, and interpretation.

## 1. Introduction

Rehabilitation science has increasingly emphasized the importance of clearly specifying how interventions are expected to produce change. Frameworks such as the Rehabilitation Treatment Specification System (RTSS) [1] and the Reporting GUideline for Intervention DEscription in Rehabilitation (GUIDE-Rehab) [2] aim to increase research translation and replication by requiring explicit linkage between intervention ingredients, mechanisms of action, and measurable targets [3,4]. Despite these advances, interventions grounded in developmental and neurobehavioral theories, including Ayres Sensory Integration^®^ (ASI) intervention [5,6,7], remain difficult to interpret within these frameworks. ASI intervention represents a useful test case because it is widely implemented, theoretically rich, and frequently evaluated using participation-level outcomes. This paper examines how ASI intervention can be conceptualized within RTSS and GUIDE-Rehab. We argue that Ayres’ theory of sensory integration functions primarily as a treatment theory for understanding how ASI intervention is expected to produce change in proximal sensory–motor capacities, whereas the effects of ASI intervention on participation may be best understood through enablement theory. This distinction clarifies persistent ambiguities in the literature and supports more precise intervention specification and evaluation. We further integrate data-driven decision making (DDDM) to clarify how assessment, hypothesis generation, and intervention planning align within this framework.

## 2. Why Sensory Integration Is Difficulty to Specify

Theories and interventions related to the integration and processing of sensory information have evolved across decades and disciplines, resulting in variability in terminology, application, and interpretation. The theory of sensory integration was originally conceptualized and described by Dr. A. Jean Ayres [5,6,7] who sought to link observable behavior with underlying neural mechanisms related to sensory processing and integration. Drawing on her training in occupational therapy, neuroscience, and educational psychology, Ayres proposed relationships between central nervous system processes and functional behavioral and learning outcomes [8].

In applying RTSS to ASI, we distinguish Ayres’ original ideas from the explanations offered by contemporary neuroscience. Ayres proposed that the nervous system could change through experience and that, even when difficulties were present, the brain could reorganize and function more efficiently. She suggested that this capacity could explain lasting changes following intervention [6] (p. 20). Contemporary research builds on this idea by describing the structural, cellular, and molecular changes that may support it [9,10]. Ayres also emphasized the importance of neural inhibition and interactions between higher and lower brain centers in organizing sensory information [10]. Later research offers more specific explanations involving sensory gating, or filtering unnecessary sensory information; autonomic regulation, including sympathetic and parasympathetic responses; and communication within specific brain networks [9]. These explanations extend Ayres’ ideas but should not be attributed to her in their current detail. Likewise, her broad idea of increased neural efficiency differs from the specific ways researchers now measure processing and network efficiency. In this manuscript, we use RTSS to organize these original ideas and later explanations as proposed mechanisms linking ASI intervention ingredients to treatment targets. These links remain hypotheses rather than established explanations of how ASI produces change.

Ayres’ theory of sensory integration (also known as ASI theory) has had widespread influence on the development of interventions for children with neurodevelopmental disabilities, learning disorders, and behavioral differences. Ayres herself developed the intervention now known as Ayres Sensory Integration^®^ (ASI), which is grounded in the core tenets of ASI theory. ASI intervention is characterized by individualized, child-led, play-based intervention delivered in a sensory-rich environment, in which therapists carefully select and grade sensory–motor experiences (e.g., tactile, vestibular, proprioceptive input) to create a just-right challenge, support engagement, and elicit adaptive responses within a strong therapeutic alliance [11,12]. At the same time, ASI theory has informed a broader range of intervention approaches, including sensory-based strategies, behavioral self-regulation programs, and environmental modifications that do not adhere comprehensively to Ayres’ principles (see for example Bodison & Parham, 2018 [13], Piller et al., 2025 [14]). This variability has contributed to ongoing scrutiny of both Ayres’ theory of sensory integration and the interventions derived from it. For the purposes of this paper, ASI intervention refers specifically to the intervention developed by Ayres and operationalized through the ASI Fidelity Measure [11,12]. Because it was derived directly from Ayres’ theory of sensory integration, ASI intervention serves as the intervention model discussed throughout this paper.

Clarifying the distinction between theory and intervention also highlights a second conceptual challenge: the level at which Ayres’ theory of sensory integration explains change does not necessarily correspond to the level at which intervention outcomes are typically evaluated. Grounded in Ayres’ theory, ASI intervention targets proximal sensory–motor capacities and the neurophysiological processes hypothesized to support them, yet intervention outcomes are often measured at the level of participation, such as engagement in school, play, or daily routines. When participation outcomes are evaluated without explicit specification of the proximal treatment targets and mechanisms through which change is expected to occur, the hypothesized pathways linking intervention to outcome remain unclear. This limits causal inference and contributes to persistent questions regarding the effectiveness of ASI intervention.

Taken together, these issues suggest that the central challenge is not simply whether interventions based on Ayres’ theory of sensory integration are effective, but whether the relationships among theory, intervention, and outcomes have been sufficiently specified. RTSS and GUIDE-Rehab provide a structure for addressing this gap by encouraging explicit linkages among treatment ingredients, mechanisms of action, targets, and broader functional outcomes. Central to this effort is distinguishing between treatment theory, which explains how intervention ingredients are expected to produce changes in specific aspects of functioning, and enablement theory, which explains how changes in functioning may contribute to broader outcomes, including occupational performance and participation. Although these theories serve distinct explanatory functions, a single theoretical framework may encompass elements of both. Therefore, before examining how Ayres’ theory of sensory integration can be applied within the RTSS framework, we first establish the criteria for distinguishing treatment and enablement theories and consider how these distinctions inform the specification and evaluation of ASI intervention.

## 3. Distinguishing Treatment Theory from Enablement Theory

The distinction between treatment theory and enablement theory has been articulated within rehabilitation science and subsequently incorporated into the RTSS [1,15,16]. Treatment theory explains how specific intervention ingredients are expected to produce changes in defined aspects of functioning. Within the RTSS, treatment theory is characterized by three interconnected elements: (1) identifiable *treatment ingredients*, representing the actions or materials provided by the clinician; (2) specific, measurable *treatment targets*, representing the proximal changes the intervention is intended to produce; and (3) known or hypothesized *mechanisms of action* that explain how the ingredients produce changes in those targets.

Enablement theory, in contrast, explains the relationships among different aspects of functioning and how changes in one domain may contribute to changes in another. These relationships may involve body functions, activities, participation, personal factors, and environmental conditions. Rather than specifying how intervention ingredients directly produce change, enablement theory identifies the pathways through which changes in functioning are hypothesized to influence broader participation aims. Importantly, enablement theory can also guide the selection of treatment targets by identifying which proximal capacities or contextual factors are expected to contribute to a desired participation outcome. Key differences between treatment and enablement theory can be found in Table 1.

For the purposes of this paper, we apply these established distinctions to examine the explanatory functions of Ayres’ theory of sensory integration. Specifically, we consider whether its theoretical propositions explain (1) how intervention ingredients are expected to produce changes in proximal sensory–motor capacities, consistent with treatment theory, or (2) how changes in those capacities are expected to influence occupational performance and participation, consistent with enablement theory. These classifications are not necessarily mutually exclusive, as a broader theoretical framework may serve both functions depending on the relationships being explained. Our analysis therefore concerns how Ayres’ theory functions within the specified intervention pathway rather than assigning it to a single, exclusive theoretical category.

## 4. Sensory Integration as Treatment Theory

According to Ayres’ theory of sensory integration, difficulties in the central nervous system’s integration and processing of sensory information are hypothesized to contribute to challenges in praxis, bilateral coordination, body scheme, postural control, sensory modulation, and sensory discrimination, which in turn influence functional and adaptive behavior [8]. When applied to intervention, a central theoretical assumption is that providing individualized, graded sensory–motor experiences that facilitate more effective processing and integration of vestibular, proprioceptive, and tactile sensory information supports improvements in these proximal sensory–motor capacities. These proposed relationships between intervention ingredients and proximal targets provide the theoretical foundation for conceptualizing ASI primarily as a treatment theory.

Within the RTSS framework, treatment theory specifies how the actions of the clinician (i.e., treatment ingredients) are expected to produce direct changes in the client (i.e., treatment targets). Treatment theory therefore provides the rationale for selecting particular ingredients to address a specific target, with mechanisms of action describing the processes through which those ingredients are expected to produce the intended change [1]. Grounded in Ayres’ theory of sensory integration, ASI intervention includes therapist actions that intentionally select, structure, and grade sensory–motor experiences involving vestibular, proprioceptive, tactile, and other sensory modalities. Within the RTSS framework, these therapist actions constitute the treatment ingredients. Importantly, therapist actions that dynamically adjust task demands, environmental conditions, and sensory input in response to the individual’s performance are also essential treatment ingredients within the RTSS framework.

The primary treatment targets of ASI intervention, as defined within the RTSS, are proximal, measurable aspects of functioning that reflect the underlying capacities hypothesized to support adaptive behavior [1]. Targets of ASI intervention include sensory discrimination (e.g., the ability to detect and differentiate sensory input including the awareness of one’s own body in space), sensory modulation (e.g., the regulation of responses to sensory stimuli), postural control (e.g., the ability to maintain and adjust body position in response to sensory input), and praxis (e.g., the planning and execution of novel motor actions including bilateral motor tasks). These treatment targets represent specific, modifiable constructs that are directly influenced by ASI intervention and can be operationalized through assessment and outcome measurement. Importantly, they are distinguished from participation-level outcomes, which are not direct targets of ASI intervention but are hypothesized to improve through changes in these underlying capacities [7].

The mechanisms of action underlying ASI intervention, based on Ayres’ theory of sensory integration, are hypothesized neurodevelopmental and neurophysiological processes that explain how therapist-delivered treatment ingredients are expected to produce change in defined treatment targets. Specifically, ASI intervention is hypothesized to facilitate more effective detection, discrimination, and integration of sensory information through repeated, meaningful sensory–motor experiences, thereby supporting more efficient organization of neural responses. These processes are further expected to enhance anticipatory postural control, improve the coordination of sensory information to support adaptive action, and facilitate the generation of adaptive responses within increasingly complex contexts. Within the RTSS framework, experience-dependent neuroplasticity and more efficient and accurate central nervous system (CNS) integration of sensory input are conceptualized not as broad explanatory concepts, but as mechanisms through which therapist-delivered treatment ingredients are hypothesized to produce measurable change in defined treatment targets (e.g., sensory discrimination, sensory modulation, postural control, and praxis). Table 2 presents representative treatment ingredients derived from Ayres’ theory of sensory integration.

Ayres’ theory of sensory integration can therefore be conceptualized as a bottom-up neurodevelopmental treatment theory in which therapist-delivered sensory–motor experiences are hypothesized to produce change in foundational sensory and sensorimotor capacities through experience-dependent neural mechanisms. This framing aligns with the RTSS by explicitly linking treatment ingredients, hypothesized neural mechanisms of action, and proximal treatment targets, thereby providing a framework for testing the mechanistic pathways underlying ASI intervention.

## 5. Sensory Integration and Enablement Theory

Within the RTSS framework, treatment theory explains how therapist-delivered treatment ingredients produce changes in proximal treatment targets, whereas enablement theory explains how changes in treatment targets contribute to more distal functional and participation outcomes, which the RTSS refers to as aims. While we argue that Ayres’ theory of sensory integration should function primarily as a treatment theory, her work extended beyond proximal sensory and sensorimotor functions. Her theoretical postulates proposed that improved sensory integration could contribute to changes in adaptive behavior, learning, and participation in everyday activities through mechanisms of neural development and neural plasticity [10]. From this perspective, Ayres’ theory of sensory integration may itself be conceptualized as an enablement theory when it is used to explain the full pathway from changes in sensory integration functions to participation-level outcomes. This conceptualization is most appropriate when ASI theory is the primary theoretical framework used to explain how change occurs across that entire pathway.

In practice, however, participation is unlikely to be explained by changes in sensory and sensorimotor capacities alone. Participation emerges through interactions among multiple personal capacities, task demands, environmental conditions, and motivational factors such as volition and self-efficacy [15,18]. Broader enablement theories may therefore provide a more comprehensive framework for explaining how changes in ASI treatment targets combine with other influences to support participation.

Applied to ASI intervention, for example, improvements in proximal treatment targets such as sensory discrimination, postural control, or praxis may increase a child’s capacity to participate in a desired activity but may not, by themselves, produce changes in participation. Whether increased capacity translates into participation may also depend on the child’s motivation and self-efficacy, opportunities for practice, caregiver characteristics, characteristics of the activity, and environmental supports or barriers.

### 5.1. An Illustrative Case

We provide the following case of M.J. to illustrate how treatment and enablement theories can be used in various ways depending on the intention and clinical reasoning process of the treating clinician.

**Figure 1:**
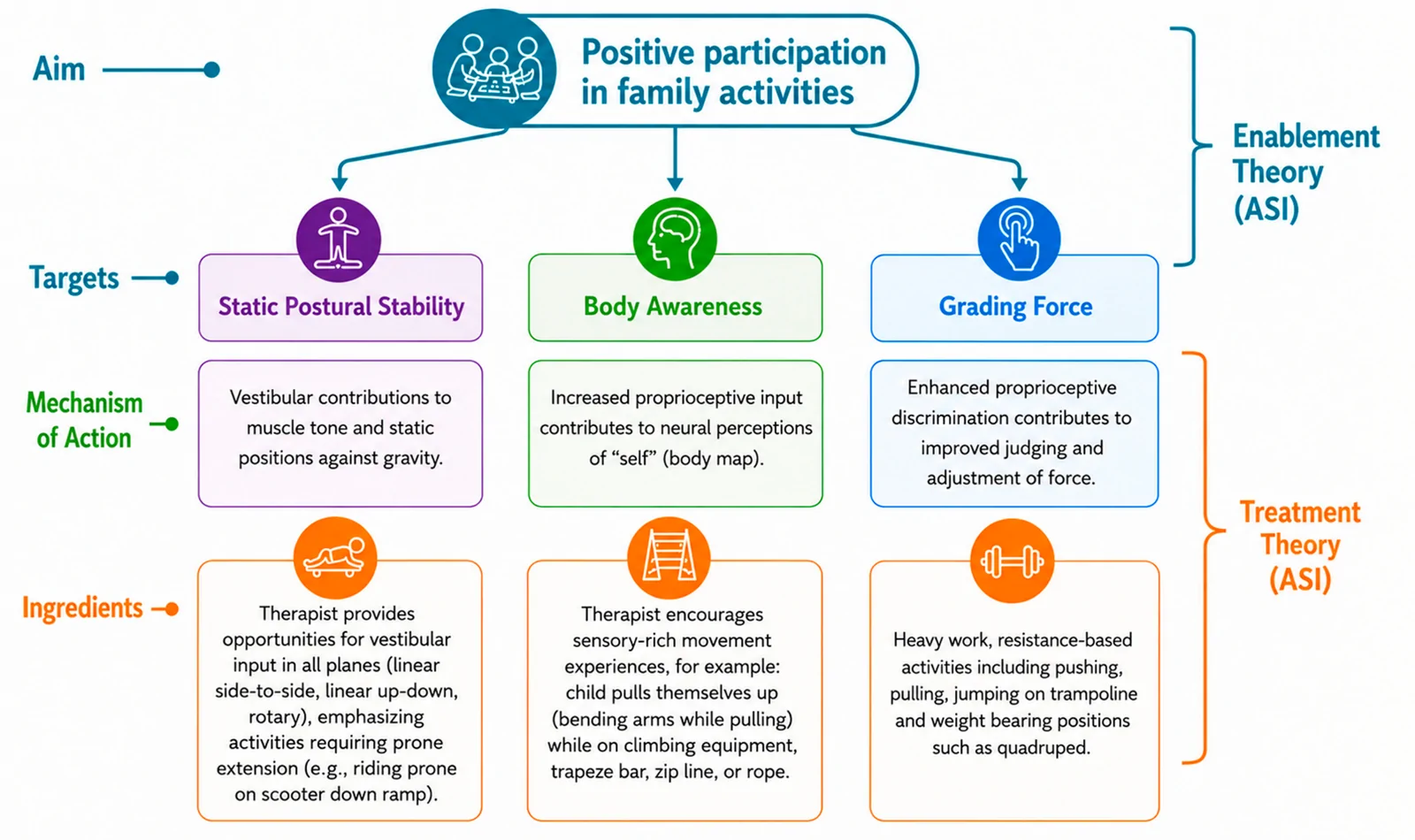
Example of ASI as both Treatment and Enablement Theory. Note: ASI = Ayres theory of Sensory Integration.

**Case.** M.J. is an 8-year-old child with sensory-based dyspraxia characterized by low muscle tone, reduced tactile and proprioceptive body awareness, and poor bilateral coordination. M.J. also has a Social (Pragmatic) Communication Disorder and is receiving speech–language therapy in addition to occupational therapy. M.J.’s family identified positive participation in family activities, particularly games and sports, as an important participation-level goal. In one scenario, presented in Figure 1, an occupational therapy clinician may hypothesize that changes in proximal targets such as static postural stability, body awareness, and force grading will lead to changes in participation using Ayres’ theory of sensory integration as an enablement theory. Because the ingredients to change the identified targets are also based on Ayres’ theory of sensory integration, Figure 1 represents a situation in which Ayres SI theory operates as both treatment and enablement theory.

**Figure 2:**
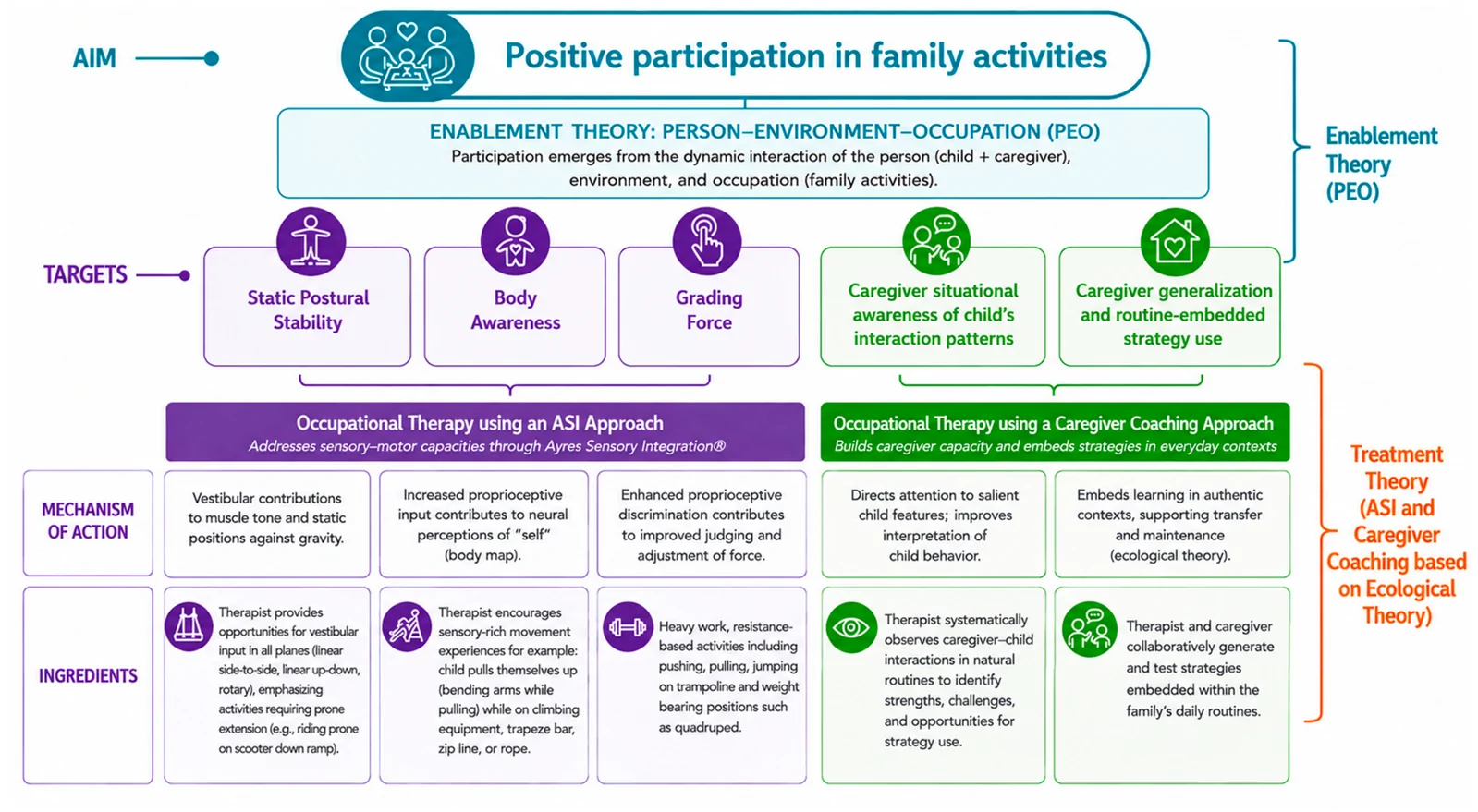
Example of Multiple Treatment and Enablement Theories Applied across Occupational Therapy. Note: PEO = Person–Environment–Occupation; ASI = Ayres theory of Sensory Integration.

An alternative scenario is one in which the clinician hypothesizes that changes in proximal sensory–motor capacities alone may be insufficient to achieve or generalize participation outcomes. In this scenario, presented in Figure 2, the occupational therapy clinician applies two distinct treatment theories, Ayres Sensory Integration theory and caregiver coaching based on ecological theory, to address different treatment targets. ASI intervention targets postural stability, body awareness, and force grading, while caregiver coaching targets caregiver understanding of the child’s needs and use of strategies within family routines [19]. The Person–Environment–Occupation (PEO) model serves as the overarching enablement theory, explaining how changes in these treatment targets interact with personal, environmental, and occupational factors to support participation in family activities [20].

**Figure 3:**
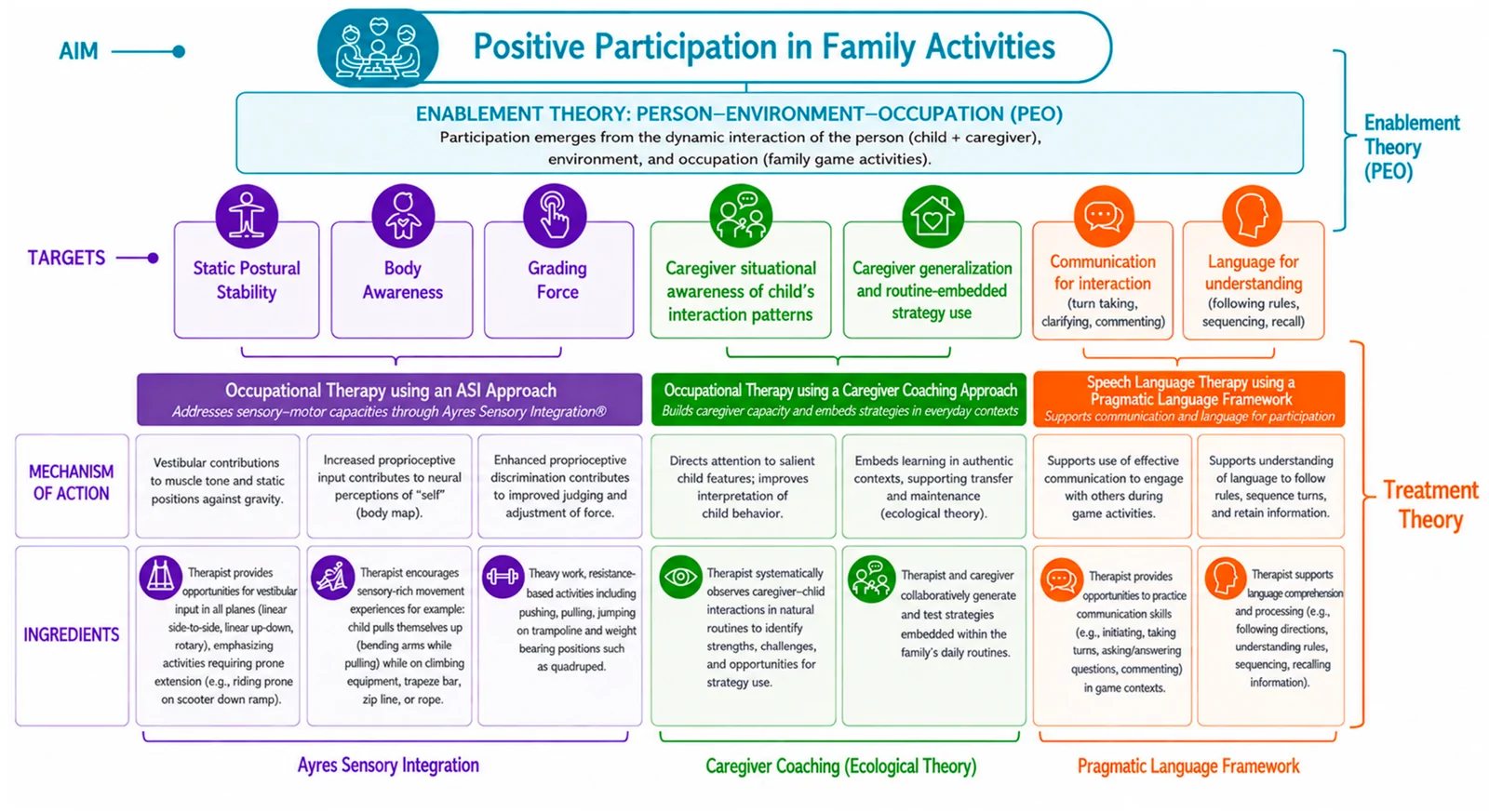
Example of Multiple Treatment and Enablement Theories Applied across Occupational Therapy and Speech–Language Pathology. Note: PEO = Person–Environment–Occupation; ASI = Ayres theory of Sensory Integration.

Finally, Figure 3 extends this framework to a multidisciplinary scenario in which speech–language therapy addresses additional proximal targets related to communication and language. The RTSS provides a common structure for specifying the distinct interventions and their targets, while PEO remains the overarching enablement framework linking changes across these targets and other contextual influences to the shared participation aim.

### 5.2. Treatment and Enablement Summary

Based on the scenarios presented, we argue that Ayres’ theory of sensory integration most clearly serves as a treatment theory, with the potential to serve enablement functions, consistent with the breadth of Ayres’ original theoretical propositions. This is because although Ayres proposed that changes in foundational sensory and sensorimotor capacities could contribute to adaptive behavior, learning, and participation in everyday life [6], the pathways through which these changes support participation were less explicitly specified. Contemporary enablement theories provide greater explanatory flexibility by recognizing that changes in sensory and sensorimotor capacities represent only some of the factors that may contribute to participation and that their influence occurs in interaction with other personal factors, task demands, and environmental conditions [20,21,22].

Importantly, the RTSS does not adjudicate which theoretical formulation is correct or prescribe which treatment or enablement theory should be used. Rather, it requires clinicians and researchers to explicitly specify the theoretical reasoning underlying their intervention decisions and the proposed pathways from treatment ingredients to targets and, ultimately, functional outcomes. Making these assumptions explicit enhances transparency, facilitates evaluation of the proposed causal pathways, and supports more precise replication of interventions across research and practice.

## 6. Resolving Inconsistencies in the SI Literature

A persistent issue facing rehabilitation disciplines is the phenomenon that has been termed the “black box of rehabilitation” [23] (p. 1)—a phenomenon in which patient deficits and outcomes are generally well described in the literature, but the rehabilitation treatments themselves are poorly defined, making it hard to know which specific active ingredients actually cause patient recovery. As noted in a 2023 editorial in the *American Journal of Occupational Therapy*, “By failing to clearly articulate the essential characteristics, elements, and clinical reasoning processes that underlie complex and multifaceted rehabilitation interventions, a barrier is created by which research papers cannot be used by clinicians to make good clinical decisions or replicate the intervention in practice” [3] (p. 1–2).

In a 2024 scoping review, Choudhury and colleagues [24] found that only 11% of published ASI intervention studies included all recommended elements of treatment specificity (i.e., ingredients, targets, and mechanisms of action). Although studies have reported improvements in individualized occupational performance and participation goals, particularly among autistic children receiving ASI intervention delivered with attention to fidelity [25,26,27,28], much of this literature retains a “black box” problem. Specifically, favorable participation-level outcomes, frequently measured using Goal Attainment Scaling, are not always accompanied by clearly documented changes in proximal treatment targets or evidence of the neural and behavioral mechanisms hypothesized to produce those changes. Without greater specificity regarding these pathways, it remains difficult to determine why ASI intervention works, for whom, and under what conditions, potentially limiting its replication, refinement, and translation into practice. Greater treatment specificity provides an opportunity to move beyond documenting whether change occurred to testing whether it occurred through hypothesized pathways. Explicit specification of treatment ingredients, intended proximal targets, and proposed mechanisms of action can guide the development of testable mechanistic hypotheses and the selection of neural, behavioral, and clinical measures needed to evaluate them.

This specificity is particularly important for advancing mechanistic research. ASI treatment ingredients are theorized to alter sensory and sensorimotor functioning through experience-dependent changes in neural processing and organization; however, the specific neural mechanisms linking particular ingredients to particular treatment targets remain incompletely specified and tested. Explicit treatment theory can identify hypothesized causal pathways; for example, which features of sensory–motor experience are expected to engage particular neural processes, how changes in those processes are expected to produce change in a proximal sensory or sensorimotor target, and which neural and behavioral measures would provide evidence for that pathway. When proximal target change is not detected, such specification also permits more informative interpretation: the target may not have changed, the selected measure may have been insufficiently sensitive, or the hypothesized neural mechanism itself may require revision. In this way, RTSS-informed specification can help transform clinical reasoning about ASI mechanisms into empirically testable hypotheses about intervention-induced neural change.

Importantly, specifying the mechanisms through which ASI intervention influences proximal treatment targets addresses only part of the pathway to participation outcomes. Participation may emerge through interactions among changes in foundational sensory and sensorimotor capacities and other influences, including personal factors, task demands, environmental supports, caregiver interactions, and opportunities for engagement. Participation may therefore improve even when changes in individual proximal treatment targets are difficult to detect or insufficient to explain the observed outcome. Explicit specification of enablement theory can make these hypothesized pathways visible, allowing researchers to distinguish neural and behavioral mechanisms of treatment-induced change from the broader processes through which those changes are translated into meaningful participation outcomes.

Distinguishing between treatment theory and enablement theory, therefore, clarifies the respective roles of each, strengthens the specification of ASI intervention, enhances clinical reasoning, and provides a common language for communicating the complexity of intervention to interdisciplinary audiences.

## 7. Specifying Outcomes Within Different Models and Frameworks

While one might see the benefits of using the RTSS or GUIDE Rehab, a barrier to implementation may be perceived misalignment with existing frameworks. In this section we argue that treatment specification can be aligned with two models commonly used in conjunction with Ayres’ Sensory Integration: The Occupational Therapy Practice Framework [29] and Data Driven Decision Making [30].

### 7.1. Treatment Specification and the Occupational Therapy Practice Framework

Within the Occupational Therapy Practice Framework (OTPF), functional outcomes represent the results of the occupational therapy process, while the occupational therapy domain includes multiple interacting aspects of functioning, including client factors, performance skills, occupational performance, and participation [29]. Although the OTPF does not distinguish these changes as targets or aims, its description of the relationships among underlying client factors, performance skills, and occupational performance is compatible with this distinction. Within RTSS, changes in client factors or performance skills may function as the direct targets of intervention, whereas changes in occupational performance and participation may represent more distal aims that are hypothesized to result from changes in those targets. Importantly, the OTPF provides the process by which occupational therapy intervention should occur, while the RTSS encourages specificity by identifying which domains are direct targets of a particular intervention and how changing targets are hypothesized to contribute to downstream outcomes.

### 7.2. Treatment Specification Within Data-Driven Decision Making

Data-Driven Decision Making (DDDM) provides another familiar clinical reasoning process within which treatment specification can be integrated. Schaaf and Mailloux (2026) distinguish between proximal outcomes, reflecting changes in sensory–motor capacities, and distal outcomes, reflecting changes in occupational performance and participation [30]. In DDDM, assessment data are used to generate hypotheses linking proximal sensory–motor factors to distal strengths and challenges in occupational performance and participation, which subsequently inform intervention planning and outcome measurement [31].

These concepts align closely with treatment specification. Proximal outcomes in DDDM may function as RTSS treatment targets when they represent the changes directly addressed by intervention, whereas distal outcomes may function as treatment aims. Treatment theory makes explicit how intervention ingredients are hypothesized to produce change in proximal targets, while enablement theory describes how changes in those targets are hypothesized to contribute to occupational performance and participation. Thus, treatment specification adds precision to relationships already represented within DDDM rather than requiring clinicians to adopt a separate clinical reasoning process.

**Figure 4:**
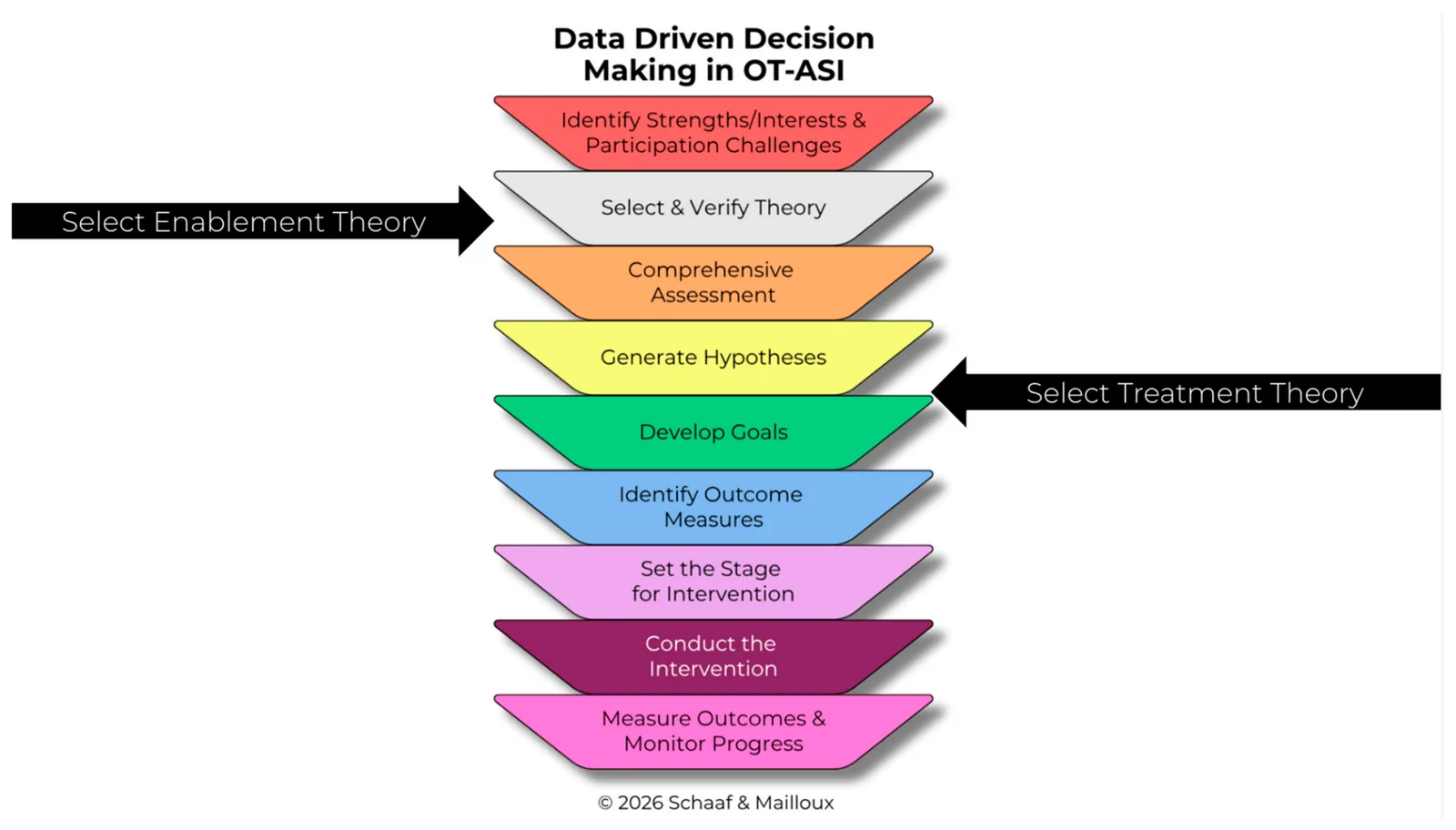
Incorporating Enablement and Treatment Theory Identification into DDDM. Note: Reprinted with permission from Schaaf, R.C. & Mailloux, Z. [32].

Figure 4 illustrates how treatment and enablement theories can be made explicit within the DDDM process. Table 3 summarizes the corresponding terminology across the OTPF, DDDM, and RTSS while Table 4 applies these concepts to the case of M.J.

## 8. Limitations

While this paper offers a conceptual framework for examining how Ayres’ theory of sensory integration can be applied within the RTSS, empirical testing is needed to evaluate its utility. The interpretation of ASI as a treatment or enablement theory depends in part on how the clinician applies the theory. As the illustrative case is a single example, it may not reflect the range of ways clinicians interpret and apply the framework. In this paper, we conceptualize ASI as functioning primarily as a treatment theory, with broader enablement models, such as PEO, providing a framework for understanding how changes in treatment targets interact with personal, environmental, and occupational factors to support participation aims.

Although ASI can also serve as an enablement theory by proposing that changes in sensory–motor capacities contribute to participation, the pathways are less explicitly specified. Even when changes in proximal treatment targets are documented, these findings alone do not establish that such changes mediate improvements in participation; testing these relationships requires appropriately designed empirical studies.

Given the multifactorial nature of participation, ASI theory alone may not fully account for the personal factors, task demands, and environmental conditions influencing occupational outcomes. Similarly, when ASI is applied as a treatment theory, the neural and behavioral mechanisms through which treatment ingredients are hypothesized to influence sensorimotor capacities, such as praxis, have not been rigorously tested. Applying ASI theory within the RTSS provides a structure for articulating the clinical reasoning underlying intervention selection and the anticipated relationships between treatment ingredients and proximal targets, but does not itself validate these proposed mechanisms.

Importantly, the absence of fully specified or empirically validated mechanistic pathways does not necessarily diminish the potential effectiveness of ASI intervention. Rather, greater specificity and transparency are needed to make the clinical reasoning underlying intervention decisions visible and testable, allowing future research to examine which treatment ingredients contribute to change, through what mechanisms, and under what conditions.

## 9. Implications for Research, Practice, and Education

Clarifying the distinction between treatment theory and enablement theory has important implications for both neuroscience and rehabilitation research. By explicitly linking therapist-delivered treatment ingredients to hypothesized neural mechanisms of action and proximal treatment targets, treatment theory provides a framework for generating and testing mechanistic hypotheses regarding how ASI intervention produces change in sensory and sensorimotor functioning. This approach may facilitate stronger integration of neuroscience with intervention research by encouraging investigators to align neurophysiological measures with specific treatment ingredients and intended treatment targets, rather than relying solely on participation-level outcomes.

For clinical practice, this framework encourages clinicians to move beyond selecting activities based primarily on experience or tradition and instead articulate the neurodevelopmental rationale underlying intervention decisions. Explicit specification of treatment ingredients, hypothesized neural mechanisms, and proximal treatment targets strengthens clinical reasoning while providing a common language for communicating intervention decisions to interdisciplinary colleagues, researchers, and families.

For education, distinguishing between treatment theory and enablement theory provides a structured framework for teaching clinical reasoning. Explicit specification of treatment ingredients hypothesized neural mechanisms, proximal treatment targets, and participation aims helps learners understand not only what intervention strategies are selected, but why they are expected to influence brain function, behavior, and participation. Making these relationships explicit may facilitate more consistent instruction, supervision, and competency development across clinicians with varying levels of experience.

Importantly, specification does not require abandonment of currently used frameworks or models such as the OTPF or DDDM. Integrating treatment specification into these existing approaches can enhance transparency and make the clinical reasoning underlying intervention decisions more explicit.

## 10. Conclusions

We propose in this paper that sensory integration theory as described by Ayres is best understood as a treatment theory targeting foundational sensory–motor capacities while its effects on participation may be more accurately interpreted through an enablement lens that emphasizes coordination and convergence of multiple factors. This distinction resolves longstanding ambiguities in the literature and aligns the approach with contemporary frameworks for treatment specification and reporting. Continued refinement of these concepts will support more transparent, rigorous, and impactful rehabilitation research and practice. Future work should focus on validating the hypothesized neural mechanisms underlying ASI intervention, refining the specification of treatment ingredients and proximal treatment targets, and identifying biomarkers and behavioral measures capable of capturing treatment-induced changes at multiple levels of the treatment pathway. Integrating treatment theory with enablement theory, implementation frameworks, and reporting guidelines such as GUIDE-Rehab may further strengthen the translation of neuroscience into rehabilitation practice.

## Figures and Tables

**Table 1:** Characteristics of Treatment and Enablement Theories in Rehabilitation.

Classification Criterion	Treatment Theory	Enablement Theory
Primary explanatory question	How and why does an intervention produce change in a treatment target?	How does change in one or more aspects of functioning influence other aspects of functioning or participation?
Primary causal relationnship	Treatment ingredients → proximal treatment targets.	Changes in treatment targets → changes in other aspects of functioning → participation outcomes.
Defining elements	Treatment ingredients, targets, and hypothesized mechanisms of action.	Domains of functioning and hypothesized causal relationships among them.
Role of intervention	Explains how specific intervention ingredients directly produce change in targeted aspects of functioning.	Explains how changes in specific aspects of functioning (targets) contribute to broader outcomes without necessarily specifying the intervention responsible for those changes.
Clinical reasoning function	Guides selection and specification of treatment ingredients to achieve desired targets.	Guides identification of treatment targets and contextual factors relevant to desired functional outcomes.
Empirical Evaluation	Tests ingredient-target relationships and hypothesized mechanisms of action.	Tests hypothesized relationships among changes in targets and downstream participation outcomes.
Application to Ayres Sensory Integration	Explains how graded sensory–motor experiences are hypothesized to change sensory modulation, discrimination, postural control, and praxis.	Explains how changes in sensory–motor capacities may contribute to occupational performance and participation.

Note. Classification characteristics are synthesized from the Rehabilitation Treatment Specification System (RTSS) [1,15,16]. The final row represents the authors’ conceptual application to Ayres Sensory Integration^®^. These classifications describe explanatory functions and are not necessarily mutually exclusive.

**Table 2:** Example Treatment Components of ASI Intervention Based on the RTSS.

Representative Treatment Ingredients (Clinician Action)	Hypothesized Neural Mechanism of Action	Representative Proximal Treatment Target
Clinician provides graded opportunities for the child to detect, localize, compare, and differentiate tactile properties of objects without vision. Clinician grades object characteristics, tactile complexity, and visual occlusion based on the child’s performance.	Repeated activation of tactile and proprioceptive pathways is hypothesized to strengthen somatosensory discrimination and multisensory integration through primary and secondary somatosensory cortex (S1/S2), posterior parietal cortex, and associated sensorimotor networks via experience-dependent neuroplasticity.	Child’s ability to detect, localize, and differentiate tactile information.
Clinician provides individualized vestibular, proprioceptive, and tactile sensory–motor experiences that elicit adaptive responses. Clinician grades the intensity, duration, predictability, and combination of sensory input based on the child’s responses.	Repeated multisensory experiences are hypothesized to improve sensory gating, autonomic regulation, and multisensory integration through brainstem, thalamic, insular, salience, and cortical networks, resulting in more efficient regulation of sensory responses.	Child’s ability to respond adaptively to sensory stimuli during daily activities.
Clinician provides sensory–motor activities requiring upright posture, balance, and equilibrium reactions. Clinician grades movement demands, base of support, speed, and environmental complexity according to the child’s performance.	Vestibular, proprioceptive, and visual information are hypothesized to be integrated more efficiently through vestibulospinal pathways, cerebellar circuits, and cortical sensorimotor networks, supporting anticipatory postural control, equilibrium reactions, and balance.	Child’s ability to maintain postural stability and automatically adjust body position during movement.
Clinician provides novel, meaningful sensory–motor play activities requiring ideation, motor planning, sequencing, and problem solving. Clinician grades task complexity and environmental demands to maintain the just-right challenge and elicit successful adaptive responses.	Multisensory integration is hypothesized to improve body schema and motor planning through interactions among somatosensory, posterior parietal, premotor, cerebellar, and frontal motor networks. Repeated adaptive responses strengthen these networks through experience-dependent neuroplasticity.	Child’s ability to plan and organize novel motor actions to achieve a desired goal.

Note: Neural mechanisms are hypothesized based on Ayres’ theory of sensory integration and contemporary neuroscience [8,9,17] and are intended to illustrate plausible mechanistic pathways linking Ayre’s Sensory Integration (ASI) intervention ingredients to proximal treatment targets. Multiple interacting neural systems likely contribute to changes in each target.

**Table 3:** Proposed Conceptual Alignment of Outcome Terminology Across RTSS, DDDM, and the Occupational Therapy Practice Framework- 4 (OTPF–4).

RTSS	DDDM	OTPF–4
Treatment aim	Distal outcome	Occupational performance and participation outcome
Treatment target	Proximal outcome	Directly targeted client factor, performance skill, performance pattern, or occupational performance component

Note. This alignment is conceptual rather than categorical. RTSS = Rehabilitation Treatment Specification System; DDDM = Data Driven Decision Making; OTPF-4 = Occupational Therapy Practice Framework 4th Edition.

**Table 4:** Application of the Proposed Terminology Alignment to M.J.’s Case.

RTSS	DDDM	OTPF–4	Application to M.J.
Treatment aim	Distal outcome	Occupational performance and participation	Positive participation in family activities, specifically games and sports
Treatment targets	Proximal outcomes	Directly targeted client factors, performance skills, and environmental supports	ASI intervention: Improved postural control, body awareness, and force grading. Caregiver coaching: Increased caregiver understanding of M.J.’s needs and use of supports during family activities. Speech–language intervention: Improved social communication skills.

Note. ASI = Ayres Sensory Integration; RTSS = Rehabilitation Treatment Specification System; DDDM = Data-Driven Decision Making; OTPF–4 = Occupational Therapy Practice Framework, 4th Edition. Treatment targets are organized according to the intervention intended to produce change. Within the overarching PEO enablement framework, changes in these targets are hypothesized to interact with personal, environmental, and task-related factors to support the shared participation aim.

## Data Availability

No new data were created or analyzed in this study. Data sharing is not applicable to this article.

## Abbreviations

The following abbreviations are used in this manuscript:

SI	Sensory Integration
RTSS	Rehabilitation Treatment Specification System
GUIDE-Rehab	Reporting Guideline for Intervention Description in Rehabilitation
DDDM	Data-driven decision making
ASI	Ayres Sensory Integration^®^
CNS	Central Nervous System
PEO	Person–Environment–Occupation
S1	Primary somatosensory cortex
S2	Secondary somatosensory cortex
OT	Occupational Therapy
